# An Analysis of and Recommendations for the Peruvian Blood Collection and Transfusion System

**DOI:** 10.16966/2471-8211.119

**Published:** 2016-04-22

**Authors:** Paul E George, Julio Vidal, Patricia J Garcia

**Affiliations:** 1 Division of Infectious Diseases, UCLA David Geffen School of Medicine, Los Angeles, California, USA; 2 National Hospital Cayetano Heredia, Lima, Peru; 3 Unit of Epidemiology, STDs and HIV, School of Public Health and Administration, Universidad Peruana Cayetano Heredia, Lima, Peru

**Keywords:** Blood transfusion, Hepatitis B virus, Hepatitis C virus, HIV, Chagas

## Abstract

**Background:**

Peru experienced a crisis in its blood collection and supply system in the mid-2000s, as contaminated blood led to several transfusion-transmitted infections (TTI), occurring in the backdrop of extremely low voluntary donation rates and a national blood supply shortage. Thus, the Peruvian Ministry of Health (MINSA) implemented a national investigation on the safety and quality of the Peruvian blood collection/transfusion network.

**Methods:**

Every Peruvian blood bank was evaluated by MINSA from 2007–2008. These evaluations consisted of an update of the national registry of blood banks and visits to each blood bank from MINSA oversight teams. Information was collected on the condition of the blood bank personnel, equipment, supplies, and practices. Further, previously-collected blood at each blood bank was randomly selected and screened for TTI-causing pathogens.

**Results:**

Uncovered in this investigation was a fragmented, under-equipped, and poorly-staffed blood collection and transfusion network, consisting of 241 independent blood banks and resulting in suboptimal allocation of resources. Further, blood with evidence of TTI-causing pathogens (including Hepatitis B, Hepatitis C, and syphilis) and set for transfusion was discovered at three separate blood banks as part of the random screening process.

**Conclusion:**

Using the successful reorganizations of national blood supply systems in other Latin American countries as examples, Peru would be well-served to form large, high-volume, regional blood collection and transfusion centers, responsible for blood collection and screening for the entire country. The small, separate blood banks would then be transformed into a network of blood transfusion centers, not responsible for blood collection. This reorganization would allow Peru to better utilize its resources, standardize the blood collection and transfusion process, and increase voluntary donation, resulting in a safer, more abundant national blood product.

## Introduction

Globally, over 100 million units of blood are donated each year [1]. Unfortunately, in Peru there is a blood shortage. According to the Peruvian Ministry of Health (MINSA), Peru has an internal demand of 600,000 units of blood per year; however, in 2013 only 185,000 units, 30% of the need, were collected [2]. This lack of blood supply is more serious in Peru than in other Latin American countries, with Peru having an availability of eight units per 1000 inhabitants (compared to Colombia, Chile, and Ecuador who have availabilities of 15, 13, and 13 respectively) [3]. Further, only 5% of collected blood in Peru comes from the safest sources, altruistic (voluntary, unpaid) donors, which is a dangerously low number even in comparison to other Latin American countries [4,5]. Because all blood transfusions, but especially those from replacement donors (compulsory donors on behalf of a patient who needs blood), carry inherent infectious risks, it is critical to have high quality blood collection systems and screening measures as part of a national blood collection system [6].

The first blood transfusion center in Peru was established in 1929 in the San Bartolome Military Hospital; since that time Peruvian blood banks have been located inside hospitals or clinics. In fact, the only free standing blood bank was eventually incorporated into a large hospital center [7]. As such, Peruvian blood banks have been managed and overseen by their individual hospitals with minimal outside oversight. However, the National Program of Hemotherapy and Blood Banks (PRONAHEBAS) was created in 1995 under Law No. 26454, declaring as a national interest the procurement, donation, storage, transfusion and supply of human blood. PRONAHEBAS’ function is to regulate, monitor and evaluate Peruvian blood banks. Nevertheless, the implementation of regulation has been difficult as Peru does not have a cohesive national blood bank service.

Peruvian blood banks are classified as Type I or Type II. Type I are authorized to receive blood from other blood banks and transfuse this blood; however, they cannot collect their own blood. Type II are authorized to both collect blood from donors and transfuse the collected blood. Peruvian blood banks are also classified as either private or public. The private sector is small, covering less than 10% of the population [8]. The public system is divided into MINSA, which covers between 60–70% of population, EsSALUD (20–25%) and FFAA-PNP (2%). EsSALUD serves the tax-paying workers/retirees and their families and FFAA-PNP serve the Armed Forces and Police. MINSA facilities are used by the remainder of the population and include poor, unemployed, and self-employed workers.

In 2004, seven newborns in Lima, Peru were infected with transfusion-related HIV [9]. As a result, *Resolución Ministerial N° 466-2005/MINSA* was enacted, which put the National Institute of Health in charge of carrying out the External Program of Performance Evaluation of Blood Banks (PEVED) [10]. As part of this investigation, blood panels containing ten different blood samples that were positive and negative for the seven different immunological markers (Hepatitis B virus, Hepatitis C virus, Chagas, syphilis, HIV 1–2, HTLV I/II) were sent to blood banks throughout the country; the blood banks were then to screen the panels in a routine fashion. Results were poor, as only 28% of blood samples were screened accurately (i.e., no false negative or false positive). Further, in 2007, another case of transfusion-associated HIV occurred in Lima [11].

These events led to the Peruvian government declaring a national state of emergency (*Decreto Supremo N° 009-2007-SA*) in the Network of Centers of Hemotherapy and Blood Banks, leading to a nation-wide investigation of blood banks [12]. The results of these evaluations and recommendations for future blood bank oversight and management on a national level are presented in this paper.

## Methods

The first step in assessing the Peruvian blood collection system consisted of updating the national registry of blood banks, which had not been done since 1999. Each blood bank and health establishment that provided blood transfusion or collection services was required to register themselves within the national registry of blood banks, listing themselves as either Type I (transfusion only) or Type II (collection and transfusion).

Following the update of the registry, supervision teams were created to visit both public and private blood banks. The supervision teams consisted of a physician, a biologist/medical technologist, and a MINSA representative. The teams used an instrument with questionnaires based on national and WHO guidelines and expert opinions that included the following topics: organization/management of the blood bank, infrastructure, equipment, human resources, and testing supplies and reagents [13]. These visits also included a thorough revision and documentation of the state of each blood bank, with each visit requiring an advance notice to the blood bank in question and the presence of the heads of the blood banks and their associated institutions. This information was collected by the study team during the official visits and stored in a central database for monitoring, analysis, and future presentation. All visits required an advance notice to the blood bank in question and also required the presence of the heads of the blood banks and their associated institutions.

There were two visits to each blood bank. In the initial visit, official documentation and instructions for the completion of the screening process were left at each blood bank. In the final visit, the study team would return, complete the surveys, and leave in every establishment a visible sticker that defined the condition of the establishment. All information was entered into a database, whose analysis would compromise the final report. Establishments that did not comply with the minimum requirements according to the national guidelines were given seven days to rectify the situation and PRONAHEBAS was informed about the results for corrective actions.

As part of the inspection, blood samples were taken from randomly selected blood bags previously screened and ready to be transfused at all Type II blood banks. The samples were taken to the National Institute of Health to be re-tested for the seven diseases blood banks must screen for by law in Peru, including Hepatitis B virus, Hepatitis C virus, Chagas, Syphilis, HIV 1–2, HTLV I/II. All visits and sampling took place between 2007 and 2008 (Table 1).

## Results

The new national registry included a total of 241 blood banks throughout Peru, with 38% located in Lima/Callao (which represents one third of the country’s population). The majority were part of the public sector (77% in total, with 50% MINSA, 23% EsSalud, and 4% FFAA-PNP hospitals).

All blood banks in the registry were visited (Table 2). Only 24% of Type I and 80% of Type II blood banks had implemented a quality assurance system. Further, there was a shortage of proper equipment. For example, 40% of Type I blood banks did not have a refrigerator that met standards for storing blood products and 54% of Type II blood banks did not had a platelet rotator, which is necessary for storing platelets [14]. Also, many blood banks had substandard screening kits. 637 screening kits for HIV, Hepatitis B and C, HTLV, and syphilis were examined: 2% (11/637) of reagents were expired and 3% (22/637) of kits lacked the necessary reagents.

Peruvian blood banks lacked uniformity. Focusing on pricing, the cost of collection of a unit of blood ranged from 10 to 165 Peruvian soles ($3.33–$55.00 USD) and the cost of infection screening ranged from 22 to 585 soles ($7.30–$195 USD). There is no central procurement for diagnostic tests in Peru, so each blood bank buys their diagnostic kits for screening from different manufacturers, leading to vastly different costs. This cost variability is passed onto the patients, with the cost to receive a unit of blood ranging from 35 to 570 soles ($11.70–$190.00 USD). In one institution, the patient is even charged to voluntarily donate blood. Further, there is much variability even in the same region and sector; for example, there are two blood banks in one province, both belonging to the public sector, but one to MINSA and the other EsSalud. The patients who are treated by MINSA payed 65 soles ($21.70 USD) per unit of blood, where as EsSalud reported a cost of 519 soles ($173 USD) for theoretically the same product. Besides pricing, the actual reagents used for screening of blood varied. In total, the 90 Type II blood banks visited used 14 different types of HIV tests, 19 Hepatitis C tests, 11 HTLV tests, and 19 different syphilis tests (Table 3).

In the month prior to the visits, the blood banks of Peru collected a total of 16,524 units of blood; the public sector collected 96% (15,835) versus 4% (689) in the private sector and 72% (11,972) were collected in Lima/Callao versus 28% (4452) in the provinces. An interesting finding was that many blood banks were discarding units due to expiration despite the national blood supply shortage. In total, 454 units (3% of all collected blood) were discarded over the month: 83% (376) by the public sector and 17% (78) by the private sector, and 70% (318) from Lima/Callao and 30% (136) from the provinces.

### Screening of previously collected blood samples

5780 screening tests for Chagas, Hepatitis B, Hepatitis C, HTLV, syphilis and HIV were performed at the National Institute of Health on blood previously screened at Type II blood banks and ready to be transfused. Of the 835 units tested, three (0.35%) tested positive for immunological markers consistent with blood not fit for transfusion (e.g., blood that should have been discarded with proper screening), including one for syphilis, one for Hepatitis C antibodies, and one for Hepatitis B antibodies.

One unit of blood tested positive for syphilis, which did not correspond with the blood bank’s original report - the unit of blood was reported as having tested negative for all immunological markers. The blood bank was notified and confirmatory testing including RPR, ELISA, and TPHA returned positive. Given these findings, the blood bank in question was re-classified from Type II to Type I.

A unit of blood from another blood bank tested positive for antibodies against Hepatitis C in screening and confirmatory testing. The blood bank was notified immediately and the study team visited the blood bank. However, upon arrival the team was informed that the blood bank had removed one sample of blood from the positive unit and subsequently discarded the rest of the unit. Confirmatory testing on this sample returned one positive and one negative result. Because the original bag had been discarded, no further testing was able to be performed on the blood sample so the cause of the discordant results was not identified.

A unit of blood from a third blood bank tested positive for anti-HBc (Hepatitis B core) antibodies. The blood bank was notified and informed the study team that the blood screening was done by an outside laboratory. It was found that the outside laboratory had correctly marked the sample as positive for anti-HBc and the results had been interpreted incorrectly by the blood bank. The blood bank was re-classified from Type II to I. In total, 12 Type II blood banks were reclassified to Type I, either for discordant screening results or lack of proper equipment and/or infrastructure.

## Discussion

The Peruvian blood supply system is decentralized, consisting of 241 small, independent blood banks. This system is consistent with other Latin American countries in the early 2000s (for example, the number of blood banks in Argentina and Colombia was 578 and 151, respectively) and in contrast to a more centralized model (in Canada the number was 14) [4]. The results from the 2008 investigation show a lack of uniformity, equipment, and personnel that have led to missed infections in the blood supply. By creating a more centralized, standardized, and coherent blood collection system, Peru could lower costs, improve the use of human resources, equipment and tests with improved quality outcomes and as a result improve the quality of donated blood.

Centralized blood collection and processing centers utilize resources in a more efficient, cost-effective, and safe manner. A study in Mexico found that centralized blood banks are better able to establish quality control methods. As a result, the blood collected from these larger blood banks had lower prevalences of HIV, Hepatitis B, and Hepatitis C infection [15]. Conversely, small local hospital-based blood banks have been previously shown to be both wasteful and costly [16]. In Finland for example, combining blood bank services has resulted in decreased day-to-day variation in blood stock levels, simplification of the ordering process, leading to cost savings [17]. Further, the diagnostic process is not centralized in Peru, instead performed by each health establishment. For proper quality assurance, the entire process, including equipment and reagent quality, procedures, and techniques must be evaluated. However, a lack of centralization together with a weak regulatory system for diagnostic tests has resulted in the availability of a large number of brands for diagnostics of unknown quality and difficulties in establishing a quality control system for these diagnostics.

High volume centers are also more able to more efficiently and effectively train their personnel and manage blood donations [18–20]. High volume centers with centralized reference laboratories allow personnel to become more experienced [21]. Low-volume centers, for lack of experience among personnel, are more likely to have more substandard collection techniques, which increases the likelihood of adverse events such as bacterial contamination in the blood supply, which itself can cause fatal outcomes [22]. Other proven benefits of the centralization of blood collection include the proper distribution of rare blood products, fractionation of blood products, and increased turnover [21]. These improvements would minimize the amount of wasted blood products, an especially important advantage for Peru given the unfortunate combination of inadequate blood supply and a high rate of discarded blood products.

One important point is that the overall quality of a country’s blood supply depends on two factors, the quality of the blood collection/ screening system *and* the quality of the donated blood [16]. Donor recruitment and screening has previously been shown to be a precarious aspect of the Peruvian blood supply chain, with the current study mirroring those results [17]. When various organizations and high numbers of blood banks comprise national blood collection systems, the large variability in dedicated resources and poorly standardized protocols results in a deficient infrastructure for blood donor recruitment, selection, and retention, resulting in low numbers of low-risk, repeat, altruistic donors [16,23]. These low numbers of altruistic donors can be seen in countries with decentralized blood banking systems such as Peru, Bolivia (10% altruistic), and Panama (2%) [16].

Further, because Peruvian blood banks are located within and managed by hospitals/clinics, their main focus has been transfusion recipients, with few resources addressing donor recruitment. As seen in other countries, campaigns by regional blood centers have been very effective at increasing altruistic donations. In Morocco for example, regional blood centers coordinated efforts to promote voluntary blood donation, with an increase from 16% to 80% over a 20 year period [24]. By having centers that specialize in blood collection, waiting times for donation could be decreased and services offered, such as providing restrooms, drinks, and snacks, could increase, thus improving the overall blood donation experience [25].

The shortcomings of Peru’s national blood collection system are highlighted by TTIs, the high error rates in the blood panels sent to the individual laboratories, and also by the results of the screening undertaking during the national investigation. As mentioned, the study team found three (out of 810 or 0.37%) units of blood ready for transfusion to be positive for immunological markers for transmissible infections, including Hepatitis B, Hepatitis C, and syphilis. This percentage of infected units of blood is unacceptably high, as studies in other settings have shown risks of TTIs to be as much as 600 times lower [26].

Importantly, Peru does not currently have national hemovigilance system, which has been described as a potent tool to improve the quality and safety in blood transfusion and is defined as a set of surveillance procedures intended to collect and assess information on unexpected or undesirable effects resulting from the therapeutic use of blood products and to prevent their recurrence [19,27]. A hemovigilance system should cover the entire blood transfusion chain, including the donation of blood and blood components, follow-up of recipients of transfused blood, collection of data and analysis of adverse events, and the prevention of recurrent events [20,28]. Given that 29% of blood banks in Peru do not even have a register of adverse transfusion-related events, Peru would benefit from such a national system. A centralized network would facilitate the creation and maintenance of such a national system, which is already in place in many countries throughout the world [27–30].

The sub-optimal blood collection system in Peru mirrors the blood bank system of another South American country several years prior, Chile. As recently as 2007, Chile’s national blood collection system consisted of separate, low volume blood banks without a centralized system for reporting blood collection and demand [31,32]. Further, HIV sero prevalence in donated blood was high and much blood was discarded. To improve the country’s blood collection services, the Chilean Ministry of Health created a National Blood Commission which transformed the country’s blood supply chain.

This Chilean renovation consisted of several aspects. Uniform national standards and a national IT system for blood and transfusion centers were established. Importantly, blood collection began to be consolidated into three main centers. These centers became sites of modernization of IT systems, data collection, and mobile blood collection campaigns. Results have been promising, with an increase in altruistic donations from 8% to 22% and a decrease in HIV sero prevalence in collected blood from 0.04% to 0.02% [31].

Nicaragua is another example of a country that has successfully reorganized its blood collection system. Between 2007 and 2009 Nicaragua transformed small blood banks into transfusion centers with blood collection and processing being moved to two centralized hubs, increasing its altruistic donation rate from 38% to 100%, making it one of only six American countries that depend solely on universal altruistic donation [33]. Nicaragua also saw significant increases in both blood collection and transfusion rates and significant drops in TTI markers as a result of the reorganization.

## Recommendations

A proper diagnosis of the shortcomings of a country’s blood collection and transfusion system is a necessary first step, but in order to promote change diagnosis might not be enough. Governmental agencies consistently manage competing issues for funding and attention. Thus, advocacy for change by other stakeholders including international agencies (e.g., PAHO), NGOs, patients’ organizations, journalists, and academic researchers is necessary. Based on the above findings, lessons learned and successes of other countries at implementing national blood collection and transfusion systems, the recommendations are presented below.

1. Large, regional blood collection and processing centers (hemocenters) that are well-equipped and interconnected with the rest of the country for distribution are needed. Existing lowvolume blood banks should be transformed into a network of centers specializing in the transfusion (transfusion centers) rather than collection of blood. Costs would be reduced as the numerous transfusion centers would not have to contain equipment necessary for blood collection, separation, and screening, allowing for an improved allocation of resources. A system with few non-hospital hemocenters would be able to take advantage of economies of scale, which can have significant cost reduction in the collection, processing, testing of blood products and distribution of blood to transfusion centers [16].
2. Hemocenters should then take the lead on a national level at establishing or improving other aspects of the blood collection and transfusion system. Advocacy and awareness for blood donors should be led by these centers. Campaigns for altruistic donation, which have been shown effective in other settings, should be organized and implemented by these centers [24].
3. Hemocenters should also become sites for conferences, seminars, and continuing education courses on transfusion medicine. They could also work with medical and nursing schools to increase practical awareness and training among students.
4. Standardized quality assurance (QA) and improvement programs should be created and would be facilitated by centralization. The QA should include all processes from defining and buying supplies to the assurance of proper donation and transfusion practices. Databases of adverse events should also be developed with plans for improvement and prevention of recurrent events. An institution like a National Institute of Health should be in charge of such QA systems which should be performed in a standardized and systematic way, with actions taken properly and timely according to findings. Specific training programs for hemocenter personnel should be designed and tailored to the standardized processes.

## Conclusion

The Peruvian National Institute of Health’s investigation from 2007–2008 into the nation’s blood banks demonstrate a sub-optimal blood collection and transfusion system. Unfortunately, despite this comprehensive evaluation, effectively nothing has changed in the Peruvian blood collection system in the past seven years. The recommendations of this paper, based on evidence from prior renovations of other national blood systems, focus on increasing the efficiency and safety of the Peruvian blood collection and transfusion system and others that may share the same characteristics through the consolidation of many independent, low-volume blood banks into a more centralized, collective, and collaborative network of hemocenters and transfusion centers. Through this consolidation, countries could lower costs, improve the state of equipment and tests with improved quality outcomes, and improve the quality of donated blood. Although potentially difficult to implement given that many established organizations and entities benefit from the current system, strong national leadership, similar to what has been seen in other countries undergoing national renovations to health care systems, could reorganize and ultimately improve national blood collection systems.

## Figures and Tables

**Table 1:** Describes the distribution of the 241 blood banks in Peru

Distribution of Blood Banks by Type and Region. Peru 2008			
	Blood Banks Type I	Blood Banks Type II	Total
	N (%)	N (%)	N
**Lima/Callao**	55 (59.8)	37 (40.2)	92
**Other 23 regions**	96 (64.4)	53 (35.6)	149
**Total**	151 (62.7)	90 (37.3)	241

**Table 2:** Describes the organization, infrastructure, and equipment of the blood banks in Peru, broken down by blood bank type and location

Organization, Infrastructure, and Equipment of Type I and Type II Blood Bank. Peru, January 2008								
	Blood Banks type I (N=151)				Blood Banks type II (N=90)			
	Lima/Callo n=55	%	Other regions n=96	%	Lima/Callao n=37	%	Other regions n=53	%
**Organization and Management**								
Trained Personnel	46/55	83.6	87/96	90.6	36/37	97.3	52/53	98.1
Implementation of Q/A system	16/55	29.1	20/96	20.1	35/37	94.5	37/53	69.8
Institutional Transfusion Committee	39/55	71.0	57/96	59.4	34/37	91.9	46/52	88.5
**Infrastructure**								
Area for reception of transfusion requirements and for delivering results	53/55	96.4	91/96	94.8	37/37	100.0	53/53	100.0
Area for interviewing donor and blood extraction	NA	NA	NA	NA	36/37	97.3	53/53	100.0
Area for production of hemocomponents	NA	NA	NA	NA	37/37	100.0	48/53	90.6
Area for sterilization and disposition of hemocomponents	48/55	87.3	84/96	87.5	35/37	94.5	52/53	98.1
**Equipment**								
Blood refrigerator 2°C to 6°C with temperature registry	33/55	60.0	57/94*	60.6	36/37	97.3	47/53	88.7
Blood refrigerator −20°C with temperature registry	31/55	56.4	23/92*	25.0	33/37	89.2	41/53	77.4
Platelet rotator	6/54*	11.1	11/93*	11.8	27/37	73.0	14/53	26.4
Autoclave	41/54*	76.0	76/93*	81.2	35/37	94.6	52/53	98.1
Automated ELISA equipment	NA	NA	NA	NA	21/37	56.8	14/53	26.4

**Table 3:** Describes the results of the surveys from the 90 Type II blood banks in Peru

The Donation Process of Type II Blood Banks in Peru, 2007–2008 (n=90)				
	Lima/Callao n=37	%	Other regions n=53	%
Use the PRONAHEBAS form for selection of the donor	30/37	81.1	50/53	94.3
The donor signed the informed consent	34/37	91.9	51/53	96.2
Has a database (registry) of volunteer donors	26/37	70.3	48/53	90.6
Participate in voluntary donation campaigns	20/37	54.1	41/53	77.4
Verify the donor identity with the results before extracting the blood	34/37	91.9	52/53	98.1
Charge the volunteers for donating blood	0/37	0	1/53	1.9
The institution takes on the cost of supplies for the screening and donation	28/36	77.8	50/53	94.3
Return results of screening to donors	32/37	86.5	37/53	69.8
Participates in a network of blood banks	19/35	54.3	36/51	70.6
Provide blood units to other health establishments	17/37	45.6	36/53	67.9
Have participated in the PEVED	26/37	70.3	42/51	82.4
